# Availability and Quality of Web Resources for Parents of Children With Disability: Content Analysis and Usability Study

**DOI:** 10.2196/19669

**Published:** 2020-11-10

**Authors:** Anabel Buteau-Poulin, Camille Gosselin, Andréa Bergeron-Ouellet, Jocelyne Kiss, Marie-Ève Lamontagne, Désirée Maltais, Christiane Trottier, Chantal Desmarais

**Affiliations:** 1 Rehabilitation Department Université Laval Québec, QC Canada; 2 Music Department Université Laval Québec, QC Canada; 3 Physical Education Department Université Laval Québec, QC Canada

**Keywords:** disabled child, parents, health knowledge, internet-based intervention, validation study

## Abstract

**Background:**

The internet is a valuable resource for parents of typical children, who are looking for information about their children’s growth and development and how to boost them. However, for parents of children with special needs, especially for non–English-speaking parents, there are anecdotal reports stating that specific and accurate information is not available on the internet.

**Objective:**

This study aims to describe the type of information available on the internet for French-speaking parents of children with disability as well as assess the quality of the information collected.

**Methods:**

We carried out a search of the existing relevant websites targeted at parents of children with disability. We used a validated instrument to extract structural, textual, and visual characteristics of these websites and evaluate their usability.

**Results:**

In all, 42 websites were analyzed; of these, the information had been validated by a trustworthy source in only 18 (43%) websites. Networking opportunities for parents were available in only 7 (17%) websites. Most websites provided information related to autism spectrum disorder (20/42, 42%) and learning disabilities (19/42, 45%), and only a few websites discussed other disability types such as behavorial disorders and developmental language disorders (4/42, 10% each). Community, social, and civic life (9/42, 22%); domestic life (12/42, 29%); and mobility (15/42, 36%) were the less frequently covered topics. With regard to the usability evaluation, 22 of the 42 (52%) websites received a global score <70%, whereas 20 (48%) scored ≥70%

**Conclusions:**

Although the internet is an infinite source of information, it is not necessarily actionable for parents of children with disability. Some information remains difficult to find online, and networking opportunities with other parents dealing with similar challenges are scarce.

## Introduction

Parents of children with disability, such as those with impairments, activity limitations, and participation restrictions [1] (eg, autism spectrum disorder [ASD], cerebral palsy, and Down syndrome), have a greater need for support because of the additional responsibilities related to their child’s care [2] than do parents of children with typical development [3-5]. The child’s condition may considerably affect the parents’ routines, priorities, and social network [6]. This may lead to increased stress levels [7] and emotional distress, partly due to insufficient social support [6,8]. Given these constraints, parents of children with disability tend to have greater needs for information and networking [9-11].

When parents receive the initial diagnosis of their child’s disability, they may have difficulty assimilating all the information provided by professionals, especially because of the emotional upheaval they experience [12]. Moreover, they may encounter new challenges (eg, how to bathe a growing child with paraplegia, or how to educate a teenager with cognitive impairment about certain sexually inappropriate behaviors in public) any time, but professionals may not be available at all times to advise them. This is one of the primary reasons why these parents turn to the internet for information [9,10,13,14]. However, considering the individualized profile of each family of children with disability, the information available online does not necessarily meet all their specific needs [15,16].

Such parents also often seek emotional support from other parents who have had a similar experience [9,17]; they believe maintaining a good, supportive social network helps them find tangible solutions to emerging issues as well as receive significant emotional reassurance [18,19]. This sharing of information is important to help them relieve the stress and alleviate the feeling of burden that may be associated with their challenges [15,19-21]. Parents find such networking and interaction as important as finding relevant information on the internet [22]. Although the internet could conceivably offer an opportunity for resourceful networking, to our knowledge, no study has examined this dimension of the use of internet. Moreover, given that most of the information available on the internet is in English, parents who do not read English may not always be able to readily access relevant information.

Finally, little is known about the format and characteristics of existing internet pages that parents of children with disability access. Only 62% of parents pay attention to whether the health care information available on websites is reliable [23], and only half of them consider themselves to be able to accurately assess the quality of the information found online [24]. These findings suggest that parents could blindly use any information found on the internet and might, in the worst case, endanger their child or themselves. Given that safety of the child is important and considerable amounts of knowledge is readily available about the descriptive characteristics that enhance usability of websites [25], it thus appears timely to provide an in-depth portrait of such internet resources.

Therefore, in this study, we aimed to (1) identify existing websites aimed at providing information to parents of children with disability, (2) summarize the content of such websites, and (3) evaluate the quality of information they provide.

## Methods

This study was conducted in a French-speaking region of Canada, within an integrated knowledge translation framework [26-28]. The study included an advisory committee comprising 2 parents (the knowledge holders) and 2 researchers (the emissaries who have the authority to disseminate this knowledge). The researchers delineated the research question and subsequently discussed the methodology as well as the interpretation of the results to ensure that the project fulfilled the parents’ initial needs (ie, to evaluate the quality of websites they use to access information). The question was brought forward by the parents on the advisory committee (ie, parents of children with disability) who believed that the content on the internet could be improved to address their information needs more adequately.

Accordingly, in the first step of this study, these parents suggested 10 websites in French that they accessed frequently. The following selection criteria were used to select the websites: (i) publicly available, (ii) written in French or in English, and (iii) intended for parents of children with disability, aged between 3 and 12 years (with the exception of one site, “Portail Enfance,” aimed at health professionals and students, but its content was, in our opinion, relevant and usable by parents). Websites presenting information about disabilities with a high prevalence (ie, ASD, cerebral palsy, intellectual disability, and learning disabilities) were first explored [29]. In the second round, websites were identified using common vocabulary search terms (eg, French counterparts of “autism spectrum disorder tips and tools,” and “special needs in cerebral palsy”) on Google. Finally, we also included websites that were found in the resources linked to the previously selected websites that were considered relevant and coherent with our selection criteria. We stopped searching for new websites when the structural, textual, and visual characteristics of the most recent websites identified were similar to at least one of the other already analyzed websites. In all, 42 websites were selected for the analyses.

The data extraction process aimed to collect descriptive characteristics of the websites (Table S1 in Multimedia Appendix 1). Two research assistants first extracted data independently from 10 websites and, after comparing their procedures, they wrote a manual to systematize the data extraction process. Next, the reviewers extracted data from 9 other websites to validate the manual’s documented procedure. Then, the data extraction tasks from the remaining 23 websites were equally distributed between them.

Thereafter, understandability and actionability of the website content were evaluated using the Patient Educational Materials Assessment Tool (PEMAT) [30] available online [31]. According to this tool, “understandability” refers to the ease in understanding what is written with regard to content, word choice and style, use of numbers, organization, layout and design, and use of visual aids. “Actionability” refers to the ease of putting into action the information presented or implementing it in real life. A single trained evaluator completed the evaluation of the selected websites. The evaluation results included both printable and audiovisual material versions. The scoring process was straightforward for all 26 items: for instance, “the material uses active voice” (item #5) or “the material provides a summary” (item #11). One (1) point was assigned if the item was fulfilled and zero (0) point, if not [31]. The COUNTIF function in Microsoft Excel was used to produce the statistics of the PEMAT scores from our extraction table. The minimum PEMAT score required for a website to be qualified as adequately understood and actionable was 70% [31]. Scores higher than 70% were further categorized as follows: 70%-79%, acceptably understandable and actionable; 80%-89%, easily understandable and actionable; and 90%-100%, perfectly understandable and actionable. One of the PEMAT subcriteria, that is, the presence of instructions or examples to explain how to perform calculations if any, was excluded from further analysis because no occurrence was found.

## Results

Of the 42 websites selected for the analysis, 39 (93%) contained information mostly in a textual format, whereas 32 (76%) also used a video format. Moreover, 25 (60%) of the 42 websites used visual aids but most did not considerably enrich the textual content (eg, an image of a father holding a book in his hands with his two children in their bed was placed next to a paragraph describing how to help children with sleep disturbance). The information presented in these websites had been reviewed either by professionals or a scientific or advisory committee for only 18 (43%) websites, whereas information was not verified for 8 (19%) websites. Moreover, we found that 19 (45%) of the 42 websites were updated within the last month, 5 (12%) were updated within the last 1-3 months, 5 (12%) were updated within the last 4-6 months, 1 (2%) was updated within the last 7-12 months, and 5 (12%) had not been updated in the previous year. Information about the last update was not determinable for 7 (17%) of all websites studied.

With regard to the type of disability, 20 (48%) of the 42 websites contained information about ASD and 19 (45%) had information about learning disabilities. Behavioral disorders and developmental language disorder were each discussed in 4 (10%) websites. Mental health issues, including anxiety, bipolar disorder, depression, and eating and personality disorders were discussed in 6 (14%) websites. Other disabilities such as cerebral lesion or palsy, Down syndrome, epilepsy, and spina bifida were discussed in 6 (14%) websites.

With regard to the activity domains of the International Classification of Functioning, Disability and Health, 9 domains were examined: learning and applying knowledge; general tasks and demands; communication; mobility; self-care; domestic life; interpersonal interactions and relationships; major life areas; and community, social, and civic life [32]*.* Most the websites addressed topics related to communication (35/42, 83%), learning and knowledge application (33/42, 79%), and relationships and interactions with others (31/42, 74%). Topics pertaining to community, social, and civic life (9/42, 21%); domestic life (12/42, 29%); and mobility (15/42, 36%) were not as frequently covered in the websites examined.

Most websites examined presented tips and advice (37/42, 88%), explanatory material (35/42, 83%), and references to similar or additional resources (32/42, 76%). Printable documents and testimonies were each found in 23 (55%) websites. Moreover, 14 (33%) websites included workshops; activity ideas for development of specific skills; personalized newsletters; or scientific articles, advertisements, questionnaires, and game platforms. Only 7 (17%) of the 42 websites examined offered networking opportunities such as comment boxes, chat, forums, and a “frequently asked questions” zone.

Based on the PEMAT usability evaluation scoring system, 22 (52%) websites received a global score <70%, whereas the remaining 20 (48%) received a score ≥70% (Table S2 in Multimedia Appendix 2).

**Figure 1:**
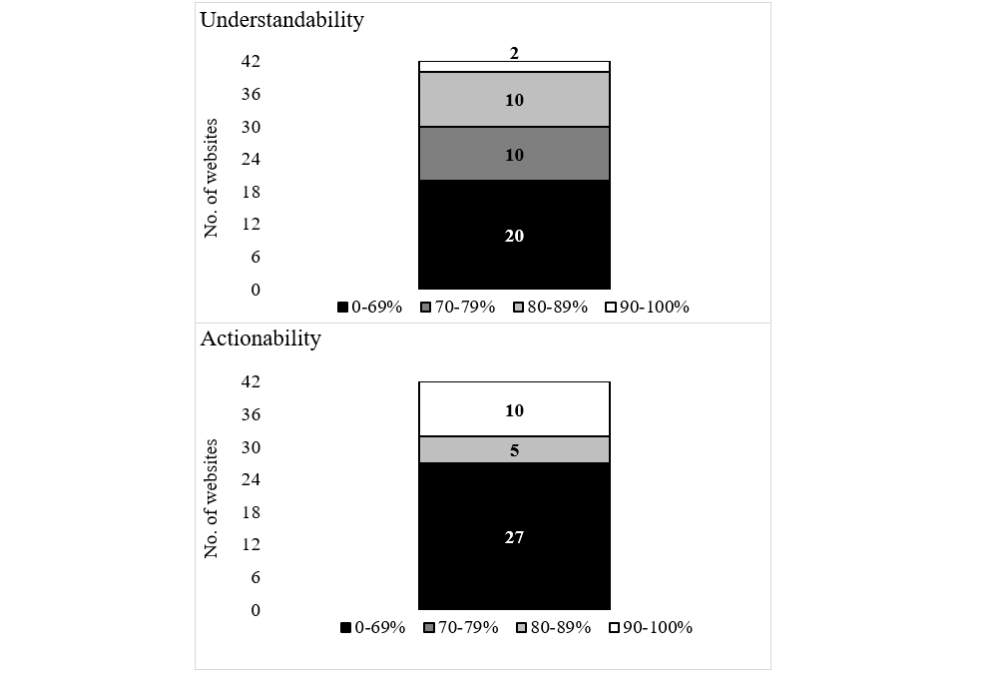
Understandability and Actionability scores based on PEMAT evaluation. PEMAT: patient educational materials assessment tool.

In the understandability category, 20 (48%) of the 42 websites (Figure 1) scored <70%, whereas 22 (52%) scored ≥70%. In the actionability category, 27 (64%) websites scored <70%, no website scored between 70% and 79%, 6 (14%) scored between 80% and 89%, and 9 (22%) scored ≥90%. When combining understandability and actionability, only 14 websites (33%) websites scored ≥70% in both categories.

**Figure 2:**
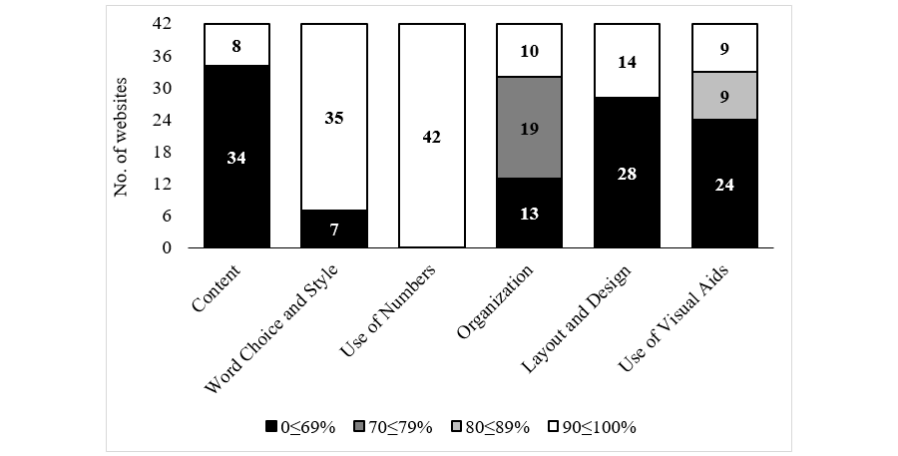
Scores per criteria in the Understandability category.

Results for the *understandability* subcriterion are presented in Figure 2, where we can see that not many websites scored over 70% for 4 aspects (content: 8/42, 19%; organization: 10/42, 24%; layout and design: 14/42, 33%; and use of visual aids: 9/14, 22%).

## Discussion

### Principal Findings

The objective of this study was to describe the type of information available on the internet for parents of children with disability as well as to assess the understandability and actionability of the web content. As a result, we recorded and studied the descriptive characteristics and PEMAT scores of 42 websites. Of these 42 websites, 39 (93%) presented information primarily in an encyclopedic textual format, which is not the most efficient format according to recently published recommendations [25], and even when websites contained visuals aids, those did not always add to the textual information.

Moreover, more than half of the websites were regarded as insufficiently understandable and actionable according to the PEMAT evaluation. These findings suggest that some parents, especially those of children with a less common disability [33], do not find adequate information on the internet that may help them address all of the child’s needs.

The type of information available on websites is mostly written (textual format) and often organized in an encyclopedic style. Although the information on these websites may be useful to the parents, the encyclopedic format is not optimal for fast and effective learning [34,35]. Similar studies have also reported that most websites rarely offer relevant visual aids to support the written information [14,22,36-38]. This finding, however, is not consistent with current technological resources that allow easy integration of multiple formats (audio, video, etc.) into websites. The websites that facilitate the most user-friendly reading experience are those that provide simple and explanatory visual aids and short texts with emphasized keywords (eg, bold characters, different character font and color, and summary boxes).

Our analysis also highlights an important message that direct access to information on the web does not necessarily imply trustable information [39,40]. In fact, approximately 57% of the websites analyzed in this study had information that was not reviewed by professionals or experts prior to publication. Nevertheless, parents searching for information or advice would not necessarily think about the trustworthiness of the source [23], or they may have difficulty determining, with certainty, which information to trust [41,42]. In such cases, people are likely to choose the information that is easier to understand or that better fits their current knowledge and understanding of the topic [43,44]. This situation is potentially problematic because there could be some risk in applying tips that are controversial. For example, a website may be promoting participation in regular physical activity but may not indicate that such activity could be dangerous for a child with epilepsy or severe osteoporosis [45]. To address this problem, we recommend that parents consult websites that publish content that has been reviewed by experts. Informational websites should also consider offering parents an online platform to discuss the content. It could be helpful for some parents to know that other parents found certain information useful and practical in a real-life situation [9,10].

Another disadvantage of more than half of the websites (55%) analyzed was that they were not frequently updated. Unavailability of up-to-date quality content was a perceived concern for 61% of the parents looking for health-related information on the internet [46]; however, other parents reported they did not verify whether the information was current or not. Lack of up-to-date content would imply that obsolete advice may be put into practice. Moreover, parents may not be able to verify information found online in a timely manner during appointments with their health professional team due to time constraints. Furthermore, parents are not necessarily prepared to address all subjects of concern during a clinical appointment [9,17,47]. The internet has thus become parents’ source of information for “pressing needs.”

More importantly, the content of the websites we analyzed mostly focused on the disability itself instead of on the child’s functioning. As a result, these websites only partially address the concerned parents’ needs and do not cover the several questions and challenges they may have [17,48]. If it were possible to search for information pertaining to various developmental disorders (eg, sleep problems), access to information that addresses parents’ needs might be easier, especially for parents of children with developmental challenges that have not yet received a definitive diagnosis. Our study findings reinforce the importance of having credible websites that are easily accessible and can be adapted to the changing needs and real-time requirements of parents of children with disability.

Finally, we also searched for websites with an added feature for opportunities for networking, but we did not find them among in the websites we examined. However, considering that technology to include such a feature on websites exists and that parents of children with disability express a pressing need for networking, it thus seems timely to consider developing such networking channels for this population.

With regard to the usability of the websites, the results of the PEMAT evaluation showed high variability among the quality of websites. The small proportion of true high scores (white: 2/42, 5%) and the large proportion of moderate (grey: 18/42, 43%) and weak scores (black: 22/42, 52%) can be interpreted as lack of relevant visual aids or clear instructions that aid the users to apply the textual information in their lives. This situation is problematic because it means that users put in place the presented advice without being sure that it is correctly executed.

### Limitations and Strengths

First, in coherence with our local context and health policies, one of the limitations of the procedures used in this study was that they were focused on Canadian resources and on French language websites. Second, our study is exploratory in nature because we did not find published recommendations for procedures to conduct the website review in a systematic manner. Thus, we proceeded in an iterative fashion, reflecting at every decisional stage and stopped the review when we concluded that we had reached content saturation.

The most important strength of this study is the patient-oriented research approach. We closely collaborated with two parents, each of whom have children with disability. This ascertains that our results are in line with the preoccupations of our target audience. Second, the juxtaposition of the PEMAT system to our data extraction table allows us to focus on the criteria that ease understandability and actionability.

### Conclusions

This study analyzed 42 existing websites on the internet intended for parents of children with disability, with an aim to determine the type of information presented, its usability, and the opportunity for networking available. Our results suggest that there is a need for websites that have expert-reviewed content, to provide accurate and accessible information at least for non–English-speaking parents of children with disability. Moreover, the results of our study highlight the need to design websites that consider usability and actionability of content or, in other words, provide opportunities for networking.

## Appendix

Multimedia Appendix 1 Descriptive characteristics extracted from the websites analyzed.

Multimedia Appendix 2 Total Patient Educational Materials Assessment Tool score for each website analyzed.

## Abbreviations

ASD: autism spectrum disorder
PEMAT: Patient Educational Materials Assessment Tool
