# Supplementary material for: Availability and Quality of Web Resources for Parents of Children With Disability: Content Analysis and Usability Study
Source: JMIR Pediatr Parent. 2020 Nov 10;3(2):e19669. doi: 10.2196/19669 (PMC7685918; doi:10.2196/19669)
Supplement: Multimedia Appendix 1 [file pediatrics_v3i2e19669_app1.pdf]

Table S1. Descriptive characteristics extracted from the websites analyzed.

Categories	Details
Name of the Website	Name of the association/organization/…
URL	Website' URL
Origin of the references	Where it has been found (Google search, hyperlink from another website, member of the research team, library resource, social media, keywords search, other)
Country	Where it comes from
Langages	In which langages information are written
Last update	When is the last time information has been added (within the last month, 1-3months, 4-6months, 7-12months, over a year)
Target audience	To whom is it addressed (caregivers, professionals, caregivers, general public, other)
Age group	For which age group is it (0-2, 3-12, >12)
Disabilities	About which disability do we find information (autism, intellectual disability, learning disabilities, developmental disorders, motor impairment, developmental language disorder (DLD), other, none/normal development)
Authors type	Who have written the information (professionals, caregivers, citizen, government, organism, association, institution, other)
Number of authors	How many authors contribute to the content (1, 2-5, 6-9, >10, many but unspecified (government website, institutions, etc.), not found)
Review and validation	Who revise information before publishing it (professionals, authors only, scientific committee, advisory committee, none, not found, other)
Contributors	Who can post information or comment on it (authors only, members of the website, general public, not found, other)
Purpose	What is the purpose/mission of the website

Content style	What kind of content is published (practical tools, tips and advices, informative material, testimonies, references to additional resource, exchange opportunities, other)
Content format	In which format content is published (textual, visual aids, video, opinion, forums, other)
ICF domains	Which ICF domain is discussed (learning and applying knowledge ; general tasks and demands ; communication ; mobility ; self care ; domestic life ; interpersonal interactions and relationships ; major life areas ; community, social and civic life)
Research tool	Is there a research tool and is it helpful
Time of navigation	How much time can we pass on the website (linked to quantity of content)
