# Supplementary material for: Availability and Quality of Web Resources for Parents of Children With Disability: Content Analysis and Usability Study
Source: JMIR Pediatr Parent. 2020 Nov 10;3(2):e19669. doi: 10.2196/19669 (PMC7685918; doi:10.2196/19669)
Supplement: Multimedia Appendix 2 [file pediatrics_v3i2e19669_app2.pdf]

Table S2. Total PEMAT score for each website analyzed.

URLs	PEMAT score (%)
https://aidersonenfant.com/	96,15
https://www.diabetesatschool.ca	96
https://naitreetgrandir.com/fr/	91,67
https://www.viedeparents.ca/	91,67
http://www.children.gov.on.ca	91,3
http://www.portailenfance.ca/wp/	91,3
http://www.alloprofparents.ca	88
https://www.healthychildren.org	88
https:/ /www.ncld.org/	88
https://www.participate-autisme.be/fr/	88
http://www.reachinginreachingout.com	88
https://www.educatout.com	87,5
http://parentspositifs.ca/triple-p-aide-relations-parents-enfants/	86,96
http://www.cheo.on.ca/fr	84
https://fantadys.com/	84
https://www.aboutkidshealth.ca	83,33
https://www.youtube.com/channel/UC6G0F26XCu9AcAbBUcGGUtw	82,35
https://www.childhooddisability.ca/fr/	81,82
http://www.vaincrelautisme.org	80
https://www.facebook.com/SOS-Nancy-coaching-familial-184211714980171/	78,26
http://www.sosnancy.com/categorie/articles/	78,26
https://www.institutta.com/troubles-apprentissage/	77,27
https://www.canchild.ca/fr	75
https://www.youtube.com/channel/UCQiIHXdMYzBNIGKwm-OfCyA/feed	75
https://canadianfamily.ca/guides/	73,91

https://www.caringforkids.cps.ca/	73,91
http://cervenad.blogspot.com	72,73
https://www.mariephilippeorthophoniste.ca/	70
https://www.facebook.com/MariePhilippeOrthophoniste/	69,57
http://www.readingrockets.org	69,57
http://www.ombresetlumiere.fr	63,64
https://www.canchild.ca/fr/diagnostics	62,5
http://www.aped.org	61,9
http://www.child- encyclopedia.com/	60,87
http://www.enfantsquebec.com	60
http://www.parentsmatter.ca	58,33
http://www.ldao.ca	57,14
http://www.autisme.qc.ca	52,63
http://parfaitemamancinglante.com	52,17
https://www.mamanpourlavie.com	39,13
https://www.aqis-iqdi.qc.ca	38,1
https://www.huddol.com/fr	36,36
